# Bowel symptoms and self-care strategies of survivors in the process of restoration after low anterior resection of rectal cancer

**DOI:** 10.1186/s12893-018-0368-5

**Published:** 2018-06-04

**Authors:** Lishi Yin, Ling Fan, Renfu Tan, Guangjing Yang, Fenglin Jiang, Chao Zhang, Jun Ma, Yang Yan, Yanhong Zou, Yaowen Zhang, Yamei Wang, Guifang Zhang

**Affiliations:** 1 Department of Hepatology, Chongqing Hospital of Traditional Chinese Medicine, Chongqing City, 400000 China; 2 Medical University of Chongqing, Chongqing City, 400000 China; 3 Nursing Department, Chongqing Hospital of Traditional Chinese Medicine, Chongqing City, 400000 China; 4 Department of Cardiovascular, Chongqing Hospital of Traditional Chinese Medicine, Chongqing City, 400000 China; 5 Department of Nephrology, Chongqing Hospital of Traditional Chinese Medicine, Chongqing City, 400000 China; 6 Science and Education Department, Chongqing Hospital of Traditional Chinese Medicine, Chongqing City, 400000 China; 7 Department of Emergency and the Intensive Care Unit, Chongqing Hospital of Traditional Chinese Medicine, No. 6 Road Panxi seven branch, Chongqing City, 400000 China

**Keywords:** Quality of life, Colorectal Cancer, Bowel symptoms, Nursing, Interview

## Abstract

**Background:**

The purpose of this research is to identify the bowel symptoms and self-care strategies for rectal cancer survivors during the recovery process following low anterior resection surgery.

**Methods:**

A total of 100 participants were investigated under the structured interview guide based on the dimensions of “symptom management theory”.

**Results:**

92% of participants reported changes in bowel habits, the most common being the frequent bowel movements and narrower stools, which we named it finger-shaped consistency stools. The 6 most frequently reported bowel symptoms were excessive flatus (93%), clustering (86%), urgency (77%), straining (62%), bowel frequency (57%) and anal pendant expansion (53%). Periodic bowel movements occurred in 19% participants. For a group of 79 participants at 6 to 24 months post-operation, 86.1% reported a significant improvement of bowel symptoms. Among 68 participants of this subgroup with significant improvements, 70.5% participants reported the length of time it took was at least 6 months. Self-care strategies adopted by participants included diet, bowel medications, practice management and exercise.

**Conclusions:**

It is necessary to educate patients on the symptoms experienced following low anterior resection surgery. Through the process of trial and error, participants have acquired self-care strategies. Healthcare professionals should learn knowledge of such strategies and help them build effective interventions.

## Background

With the overall 5-year overall survival rate of rectal cancer increasing, more and more researches focus not only on the resection of tumor but also on postoperative quality of life (QOL) after surgery. The application of stapling technique and total mesorectal excision facilitates the increased proportion of sphincter-saving surgery (SPS). The main reason for its higher rates arises from the conviction that the QOL of patients undertaking SPS is better than that of patients with a permanent stoma. But a systematic review concluded that SPS does not mean a better QOL in patients who do not require a permanent stoma [1].

Following SPS, bowel dysfunction, also called low anterior resection syndrome, is a common and trouble problem. It is characterized by frequency, urgency, incontinence, and clustering (another bowel movement within one hour of last bowel movement), affecting more than 90% of patients who undergo a low anterior resection (LAR) [2]. However, within the clinical practice, we discovered some patients experienced periodic bowel movements (i.e. stool being hard at first then mushy or liquid occurs every few days), which is not reported in previous studies [3–8]. Risk factors for developing bowel dysfunction are low-level anastomosis, end-to-end anastomosis, anastomotic leakage, acute or chronic inflammation, surgical autonomic denervation, loss of the rectal reservoir function and preoperative radiotherapy. The level of anastomosis is the most important factor [2, 9–11].

The outcome of the above bowel symptoms varied. For some patients, the symptoms can improve over time, and other patients find it difficult to treat and leave a permanent colostomy. There is scant data on the length of time it takes patients to report an improved bowel function. Because of the lack of effective medical interventions, the patients have to struggle with bowel problems for a long term. They can provide rich information for the management bowel symptoms through trial and error. Such information is vital to healthcare professionals when advising patients on the possible benefits. In 2008, Hunphreys’ teamwork developed the “symptom management theory” followed by two revisions. It is a middle-range theory, providing guidelines for symptom management [12].

The purpose of this article is to explore the bowel symptoms and self-care strategies for survivors of rectal cancer during LAR postoperative recovery using a structured interview guideline based on the “Symptom Management Theory” dimension.

## Methods

### Sample

The sample across one tertiary teaching hospital in China was chosen for the study. Including individuals were if they were 3 to 24 months after the LAR (tumors located at a maximum of 8 cm from the anal verge) for rectal cancer. The operative steps were en bloc rectal excision including (1) ligation of the inferior mesenteric artery at its origin, (2) complete mobilization of the splenic flexure, (3) transection of the proximal left colon, (4) sharp dissection in the avascular plane into the pelvis—anterior to the presacral fascia—parietal fascia and outside the fascia propria or enveloping visceral fascia, (5) division of lymphatics and middle hemorrhoidal vessels anterolaterally at the level of the pelvic floor, and (6) inclusion of all pelvic fat and lymphatic material to the level of the anorectal ring or all fat and lymphatic material at least 2 cm below the level of the distal margin. Exclusion criteria included anastomotic leakage, recurrence of rectal cancer, adjuvant radiation, pelvic exenteration, palliative care, acute or chronic inflammation and language barriers.

### Ethical consideration

Approval was gained from all the relevant ethics committees prior to the study. All interviews were conducted and documented by the same researcher. Provides individuals with information about research and documentation goals. After receiving the consent, start the interview. In the process, they can withdraw from the study at any time or refuse to answer the question.

### Data collection

The participants’ bowel symptoms and self -care strategies after surgery were investigated with the structured interview guide based on the “symptom management theory” dimensions [12]. The guide comprises symptom experience and symptom management strategies. Based upon previous data, both fixed-response and open-ended questions were included. Most of the fixed-response questions were rated on a Likert-type scale, ranging from 3 to 4 points, and sought information on the types and frequency of symptoms experienced in the past 1 month. From clinical observations, we found that some postoperative patients reported a finger-shaped consistency stools that was not shown in Bristol stool scale. Therefore, this paper added this category to this scale.

At the start of the study, most of participants were outside the hospital, and only a few returned for some reasons, such as postoperative chemotherapy and follow-up visit. With this in mind, interviews were conducted by two ways: by telephone and face to face.

### Data analysis

Data analysis was guided by the principles of deductive content analysis [13]. This analysis is used when the structure of analysis is operationalized on the basis of previous knowledge. Data were quantized by the way of frequencies and percentages as well as central tendency and dispersion.

## Results

A total of 102 patients were invited to participate in the study. Two patients refused, and finally enrolled 100 patients. 69 of the respondents participated in the telephone interview and 31 participants participated in a face-to-face interview. The effective rate is 98.0%. A sample of 100 participants (56 men) with a mean age of 60.3 ± 10.0 years was available for data. 46% participants were from rural area and 54% participants from urban area. The average interval between LAR or closure of the temporary stoma and interview was 11.9 ± 6.6 months. 21% of participants were in 3–5 months, 40% of participants were in 6–11 months and 39% of participants were in 12–24 months. The length of tumor from anal verge was 6.4 ± 1.3 cm. 75% participants had received adjuvant chemotherapy. All the participants had received both the end-to-end anastomosis and double stapled technique.

### Symptom experience

With reference to symptom experience, symptom perception, symptom evaluation and symptom responses were adapted.

### Bowel habits

92% of participants reported changes in bowel habits (Table 1). Fifty-seven percent of subjects had > 3 bowel movements / day after surgery and > 7 / day for 25% of the subjects. 19% of participants had periodic bowel movements. Almost all participants reported fewer stools after surgery. The most common (52%) fecal consistency was finger-shaped with a small amount.

**Table 1:** Bowel habits before and after surgery (*n* = 100)

Before surgery			After surgery	
	n	%	n	%
Frequency of defecation				
(1–3)times/day	44	44	23	23
(4–7)times/day	41	41	32	32
> 7times/day	12	12	25	25
(2–3)times/week	3	3	1	1
Periodic bowel movements	0	0	19	19
Stool consistency				
Hard lumps	14	14	9	9
Sausage-shaped	32	32	4	4
Finger-shaped	1	1	52	52
Soft blobs	20	20	12	12
Fluffy pieces	28	28	10	10
Liquid	5	5	1	1
Miscellaneous	0	0	12	12

### Bowel symptoms

The bowel symptoms reported are shown in Table 2. The 6 most frequently symptoms were excessive flatus, clustering, urgency, straining, bowel frequency (> 3 times/day) and anal pendant expansion. Participants reported multiple precipitating factors associated with soiling, 68 responses from 40 participants. The most frequently reported was passing wind by 34/40 (85.0%) participants, not immediate access to toilet by 24/40 (60.0%) participants, inability to consciously control bowel movements by 6/40 (15.0%) participants and physical activities by 4/40 (10.0%) participants.

**Table 2:** Bowel symptoms after surgery (*n* = 100)

	Number	Percent
Excessive flatus	93	93
Clustering	86	86
Urgency	77	77
Straining	62	62
Bowel frequency	57	57
Anal pendant expansion	53	53
Incomplete evacuation	42	42
Soiling	40	40
Perianal soreness/itching	36	36
Abdominal/rectal pain	31	31
Periodic bowel movements	19	19
Inability to distinguish between passing feces/wind	11	11

### Bowel evaluation

For a group of 79 participants 6 to 24 months postoperatively, 68 (86.1%) participants reported a significant improvement in bowel symptoms (Table 3). In this significantly improved subgroup, 48 (70.5%) participants reported the length of time it took improvement was at least 6 months.

**Table 3:** Length of time for patients reporting a significant improvement of bowel symptoms

Significant improvement	Patients at 6–11 months (*n* = 40)		Patients at 12-24 months (*n* = 39)	
	n	%	n	%
NO	7	17.5	4	10.3
YES	33		35	
3–5 months	14	35.0	6	15.4
6 months	13	32.5	16	41.0
7–11 months	6	15.0	2	5.1
12 months			9	23.1
13–24 months			2	5.1

### Social and physical responses

Restriction of leisure activities was seen in 73% participants. They avoided going out and exercised in a specific area. The availability of toilets was considered by 77% participants. 61% participants reported a sleep disturbance. For a group of 10 participants aged of ≦55 years at the 12–24 months of surgery, 7 participants continued to work and 5 of them came from rural area.

### Psychological responses

With psychological responses to open-ended questions, the deductive content analysis produced 2 categories comprising 5 subcategories (Table 4). Negative psychological responses were found in 72% participants. A secondary theme running throughout all these categories was the feeling of confidence and normality.

**Table 4:** Length of time for patients reporting a significant improvement of bowel symptoms

Significant improvement	Patients at 6–11 months (*n* = 40)		Patients at 12-24 months (*n* = 39)	
	n	%	n	%
NO	7	17.5	4	10.3
YES	33		35	
3–5 months	14	35.0	6	15.4
6 months	13	32.5	16	41.0
7-11 months	6	15.0	2	5.1
12 months			9	23.1
13–24 months			2	5.1

### Symptom management strategies

Self-care strategies adopted by participants included diet, bowel medications, practice management and exercise.

### Diet

96% of participants reported a change in diet. The change was described by high fiber, low fat, no offending food such as wine, cold beverage, reduced spicy food and stimulating food (i.e. chicken, mutton, seafood and ginger) that may induce or aggravate cancer. The 2 most common types of postsurgical diet were high fiber and low fat. In the subsequent 50-person interview, 33 (66.0%) had greasy food and 32(64.0%) participants had cold drinks developed diarrhea.

### Bowel medications

Two types of bowel medications reported by participants are presented in Table 5. More than two-fifths participants had used the medications for bowel control in the past 1 month, the most common being Imodium. Minority participants required long-term use of medications to control stool elimination. Less than two-fifths participants had used medications for perianal soreness or itching, with sitz bath being the most common reported.

**Table 5:** Bowel habits before and after surgery (*n* = 100)

Before surgery			After surgery	
	n	%	n	%
Frequency of defecation				
(1–3)times/day	44	44	23	23
(4–7)times/day	41	41	32	32
> 7times/day	12	12	25	25
(2–3)times/week	3	3	1	1
Periodic bowel movements	0	0	19	19
Stool consistency				
Hard lumps	14	14	9	9
Sausage-shaped	32	32	4	4
Finger-shaped	1	1	52	52
Soft blobs	20	20	12	12
Fluffy pieces	28	28	10	10
Liquid	5	5	1	1
Miscellaneous	0	0	12	12

### Practice management and exercise

36% of participants reported perianal soreness or itching after frequent bowel movements, which promoted them to use moist wipes and irrigation to clean the anal area. In order to prevent leakage of feces, some participants went out with spare underpants, sought the location of toilets and filled papers or pads in the anus. Physical activities were focused mainly on walking and dancing. Some participants reported a desire to defecate after meal or physical activities which were not perceived before surgery.

## Discussion

### Symptom experience

Most of participants reported a change in bowel habits with increased bowel movements and finger-shaped consistency stools. This change may be due to loss of the rectal reservoir function for the decreased ability to store feces and surgical autonomic denervation for the altered bowel motions. Periodic bowel movements reported by participants is not found in previous studies [3–8], which may be due to the rigid neorectum around the anastomosis where feces is hard to pass until a certain amount of it produces an enough pressure.

The type of bowel symptom identified in this paper is similar to the findings of earlier studies [4, 7], but its frequency rankings are higher. It could be due to the lower height of tumor resulting in poor bowel function. During the interview, we found that some participants confused the term “clustering” with “incomplete evacuation”, which may increase the frequency of the latter. A study by [4] Emmertsen and Laurberg (2012) have shown that incontinent of flatus is one of the most common bowel dysfunction, which is not identified this paper because participants did not have a try to hold flatus. Abdominal or rectal pain was a sort of dull or distending. Perianal soreness or itching was caused by bowel frequency, incomplete evacuation and intake of spicy food.

Soiling is a concern for participants. Leaking faces was mainly mushy or liquid. It was associated with the following factors: passing wind due to incomplete closure of anal canal consequent upon the internal anal sphincter dysfunction; not immediate access to toilet due to lack of control over feces resulted from the external anal sphincter weaknesses; physical activities due to the consequent increase of intra-abdominal pressure and inability to consciously control bowel movements due to the damage of transitional epithelium above dentate line. The two most common causes were passing wind and not immediate access to toilet. This is in contrast to the observations of [7] Nikoletti et al. (2008), in which physical activities and after going to the toilet were the most common.

### Bowel evaluation

Research on the time it takes patients to report improved bowel function is limited. In our study, almost all participants admitted that the bowel symptoms were improved with time. The majority of (86.1%) subjects reported a significant improvement in their bowel symptoms from 6 to 24 months, concentrating on soiling, anal pendant expansion and urgency. The most frequently reported improvements are at least 6 months. This is similar to the findings of an earlier study [14], where QOL improved over time and significantly after the first 6 months. The predictability of bowel symptoms varied. For some participants, the symptoms were predicted so that they planed evacuation at a convenient time.

### Bowel responses

More than half of participants reported bowel frequency, but less than two-fifths participants considered the extent of disturbance of leisure activities as ‘often’. This discrepancy between the symptom prevalence and the bother rating may be explained by participants’ good control of the feces, knowing the location of the toilets and sacrificing QOL. For most of participants, the consequence of resection of tumor was a small price to pay for their life. As a result, their QOL were underestimated. This phenomenon is an example of response shift discovered by Sprangers and Schwartz (1999) [15]. Five of seven participants who continued their work were from rural area, which demonstrated household income is a positive factor in ongoing work. Most (61%) participants reported a sleep disturbance due to frequency and night-time soiling and felt fatigue the next day.

Most participants had a negative psychological reaction. This may be because they did not recognize the change in bowel anatomy, did not distinguish between bowel symptoms caused by surgery and symptoms associated with cancer recurrence, there was no effective intervention. A substantial proportion of participants’ complaint their bowel function were neglected by health professionals. This is in contrast to the findings of an earlier study [16] but is supported by another study [17]. Due to lack of information support, trial and error adapted by participants for managing bowel symptoms was aimed at gaining self-confidence and normality.

### Symptom management strategies

The two most common types of diet after surgery were high fiber and low fat. Some participants followed a specific diet. Based on the principal of ensuring a nutritionally adequate diet, participants are encouraged to reduce their food intake, but with increased frequency. Almost all participants admitted that bananas, sweet potatoes and fresh vegetables facilitate stool elimination. High fiber is confusion for some participants. Depending on the type of fiber ingested, it may exacerbate problems with soft stools and evacuation. This is consistent with earlier studies [7, 18]. The two studies found that supplementation with soluble dietary fiber improves the water-holding capacity of stool solids, the consistency of stools and fecal incontinence and insoluble fiber may exacerbate diarrhea and bloating. Spicy food can cause perianal soreness, constipation and diarrhea. Due to the development of traditional Chinese medicine, some participants reported avoiding stimulating food. Norton and Chelvanayagam (2001) [19] concluded the bowel function was affected by artificial sweeteners, tea, cola drinks and chocolates, which was not confirmed in this paper.

Through interview, the current study concluded that bowel medications should be a conservative measure aimed at controlling symptoms. Three participants reported that Medilac-Vita comprising *Bacillus subtilis* sp. and *Enterococcus faecium* sp. was better than Imodium in case of frequency bowel movements and softer stool. As Western medicine failed to treat bowel problems, some participants sought help from traditional Chinese medicine. For perianal soreness, some participants reported sitz bath with water or saline solution was available and effective. Inappropriate use of bowel medications can cause unnecessary adverse effects. In this paper, some participants reported constipation and diarrhea after taking Imodium and Lactulose. Therefore, healthcare professionals should educate patients on how to use them properly.

The location and availability of toilets is necessary for this group of participants. It is encouraged to follow the successful experience of Australia to establish National Public Toilet Map and Web site [20]. Increased urgency to defecate after meal or physical activities may be experienced by some participants due to the altered bowel anatomy. Loots and Bartlett (2009) [18] encouraged participants to remain active or gradually increase their level of physical activity as the improved bowel control is associated with confidence improved. It is because more vigorous exercises such as running, swimming, and cycling may stimulate bowel activity.

### Implications for practice

Health professionals should help patients understand the nature and outcome of bowel symptoms, understand patients’ strategies for adapting to bowel symptoms, provide advice on possible benefits, and provide leadership in addressing bowel problems. Close attention should be given to patients who developed with new rectal bleeding for anastomotic recurrence (AR) of colonic cancer. AR occurred less than two years after radical resection of colon cancer. Its diagnosis can be confirmed by colonoscopy. LAR followed by intensive careful endoscopic monitoring could result in long-term disease-free survival [21].

## Conclusion

Bowel symptoms can be significantly improved for most of survivors. The most frequently reported improvement is at least 6 months. This paper identifies a new bowel symptom (i.e. periodic bowel movements). Rectal cancer survivors following low anterior resection surgery felt abandoned after surgery and lacked the information to manage bowel symptoms. Healthcare professionals should provide relevant information to support them, in particular how to discriminate between the bowel symptoms caused by surgery and the symptoms that might be associated the recurrence of cancer.

## Abbreviations

QOL: quality of life
SPS: sphincter-saving surgery
